# Popular Evidence-Based Commercial Mental Health Apps: Analysis of Engagement, Functionality, Aesthetics, and Information Quality

**DOI:** 10.2196/29689

**Published:** 2021-07-14

**Authors:** Nancy Lau, Alison O'Daffer, Joyce P Yi-Frazier, Abby R Rosenberg

**Affiliations:** 1 Palliative Care and Resilience Lab Center for Clinical and Translational Research Seattle Children’s Research Institute Seattle, WA United States; 2 Department of Psychiatry and Behavioral Sciences University of Washington School of Medicine Seattle, WA United States; 3 Cambia Palliative Care Center of Excellence University of Washington Seattle, WA United States; 4 Department of Pediatrics University of Washington School of Medicine Seattle, WA United States

**Keywords:** mobile health, mental health, behavioral health, user-centered design, evidence-based health management, smartphones, mobile phones

## Abstract

**Background:**

There is a robust market for mobile health (mHealth) apps focused on self-guided interventions to address a high prevalence of mental health disorders and behavioral health needs in the general population. Disseminating mental health interventions via mHealth technologies may help overcome barriers in access to care and has broad consumer appeal. However, development and testing of mental health apps in formal research settings are limited and far outpaced by everyday consumer use. In addition to prioritizing efficacy and effectiveness testing, researchers should examine and test app design elements that impact the user experience, increase engagement, and lead to sustained use over time.

**Objective:**

The aim of this study was to evaluate the objective and subjective quality of apps that are successful across both research and consumer sectors, and the relationships between objective app quality, subjective user ratings, and evidence-based behavior change techniques. This will help inform user-centered design considerations for mHealth researchers to maximize design elements and features associated with consumer appeal, engagement, and sustainability.

**Methods:**

We conducted a user-centered design analysis of popular consumer apps with scientific backing utilizing the well-validated Mobile Application Rating Scale (MARS). Popular consumer apps with research support were identified via a systematic search of the App Store iOS (Apple Inc) and Google Play (Google LLC) and literature review. We evaluated the quality metrics of 19 mental health apps along 4 MARS subscales, namely, Engagement, Functionality, Aesthetics, and Information Quality. MARS total and subscale scores range from 1 to 5, with higher scores representing better quality. We then extracted user ratings from app download platforms and coded apps for evidence-based treatment components. We calculated Pearson correlation coefficients to identify associations between MARS scores, App Store iOS/Google Play consumer ratings, and number of evidence-based treatment components.

**Results:**

The mean MARS score was 3.52 (SD 0.71), consumer rating was 4.22 (SD 0.54), and number of evidence-based treatment components was 2.32 (SD 1.42). Consumer ratings were significantly correlated with the MARS Functionality subscale (r=0.74, *P*<.001), Aesthetics subscale (r=0.70, *P*<.01), and total score (r=0.58, *P*=.01). Number of evidence-based intervention components was not associated with MARS scores (r=0.085, *P*=.73) or consumer ratings (r=–0.329, *P*=.16).

**Conclusions:**

In our analysis of popular research-supported consumer apps, objective app quality and subjective consumer ratings were generally high. App functionality and aesthetics were highly consistent with consumer appeal, whereas evidence-based components were not. In addition to designing treatments that work, we recommend that researchers prioritize aspects of app design that impact the user experience for engagement and sustainability (eg, ease of use, navigation, visual appeal). This will help translate evidence-based interventions to the competitive consumer app market, thus bridging the gap between research development and real-world implementation.

## Introduction

In the Digital Age, smartphones have permeated all aspects of personal and professional life. There is a robust market for mobile health (mHealth) apps focused on self-help for mental health and behavioral health needs [1,2]. Despite the widespread appeal of mHealth for mental health, we raise 2 important considerations for its adoption. First, development and testing of mental health apps in formal research settings are limited and far outpaced by everyday consumer use [2-5]. Second, app design elements such as engagement and functionality impact whether users continue to use a mobile app for sustained behavior change over time beyond initial download [6].

The aim of this study was to conduct a user-centered design analysis of the usability, engagement, and quality of popular evidence-based apps for mental health self-management utilizing the well-established Mobile Application Rating Scale (MARS) [7]. Previous publications have utilized the MARS to evaluate apps on an eclectic array of health-related topics including blood pressure, mindfulness, nutrition, diet and physical activity, deafness and hard-of-hearing, and drug–drug interactions [8-13]. This study evaluated the quality of mental health apps that are successful across both research and commercial sectors. We evaluated the relationships between objective app quality, subjective user ratings, and evidence-based behavior change techniques. This will help inform design considerations for mHealth researchers to maximize consumer appeal, engagement, and sustainability.

## Methods

### Overview

In a recent review of consumer apps, we identified 21 mental health self-management apps that were publicly available and research supported [5]. Two have since been removed by the developers, consistent with previous findings that consumer apps are retired at a rapid rate [5]. For this pool of 19 apps, we conducted the following data collection and analyses (February to April 2021) to address the current study objectives: MARS evaluations by 2 independent coders, extraction of consumer ratings from the App Store iOS (Apple Inc) and Google Play (Google LLC), coding of evidence-based treatment components, and correlation analyses.

We utilized the MARS, a validated objective measure for assessing the quality of mHealth apps [7]. The 23-item MARS provides a total score and Engagement, Functionality, Aesthetics, and Information Quality subscale scores. MARS total score and subscale scores range from 1 to 5, with higher scores representing better quality. Independent raters (NL and AO) downloaded and evaluated each app; individual item scores were averaged between raters according to accepted standards [11]. App consumer ratings were extracted and averaged across App Store iOS and Google Play. We coded app content as evidence-based behavior change techniques (ie, based in behavior change theory or psychological interventions shown to be efficacious or effective) or not evidence based (ie, digital content/modules such as daily inspirational quotes that are not a component of traditional evidence-based mental health interventions).

### Statistical Analysis

Interrater reliability was assessed using the intraclass correlation coefficient (ICC) according to established guidelines; we ran a 2-way mixed effects, average measures model with a consistency of agreement definition [14,15]. We calculated Cronbach α to assess the internal consistency of the MARS [16,17]. Descriptive statistics were used to summarize MARS scores, consumer ratings, and number of evidence-based treatment components. Pearson correlation coefficients were calculated to compare (1) the MARS overall score with consumer ratings, (2) the MARS subscale scores with consumer ratings, and (3) number of evidence-based treatment components with MARS and consumer ratings. All analyses were conducted in IBM SPSS Statistics version 27.

## Results

The MARS demonstrated high interrater reliability (ICC 0.97, 95% CI 0.97-0.98), and the total score had high internal consistency (Cronbach α=.99). Table 1 shows the MARS total score and subscale scores, mean consumer ratings obtained from app download platforms, and number of evidence-based treatment components for each of the 19 apps. The MARS total mean score for all apps was 3.52 (SD 0.71; range 2.22-4.32). The MARS subscale mean scores were as follows: Engagement, 3.98 (SD 0.82; range 2.30-4.80); Functionality, 3.42 (SD 0.80; range 2.00-4.63); Aesthetics, 3.23 (SD 0.90; range 1.67-4.67); and Information Quality, 3.47 (0.69; range 2.00-4.29).

Average consumer ratings across the App Store iOS and Google Play were 4.22 (SD 0.54; range 3.24-4.86). Average number of evidence-based treatment components was 2.32 (SD 1.42; range 1-5). Notably, 8/19 (42%) of apps consisted of a singular approach to treatment (ie, had only 1 evidence-based treatment component). Headspace had both the highest MARS total score and highest consumer rating, and is a unimodal intervention that teaches mindfulness meditation for emotion regulation and health behavior change.

**Figure 1:**
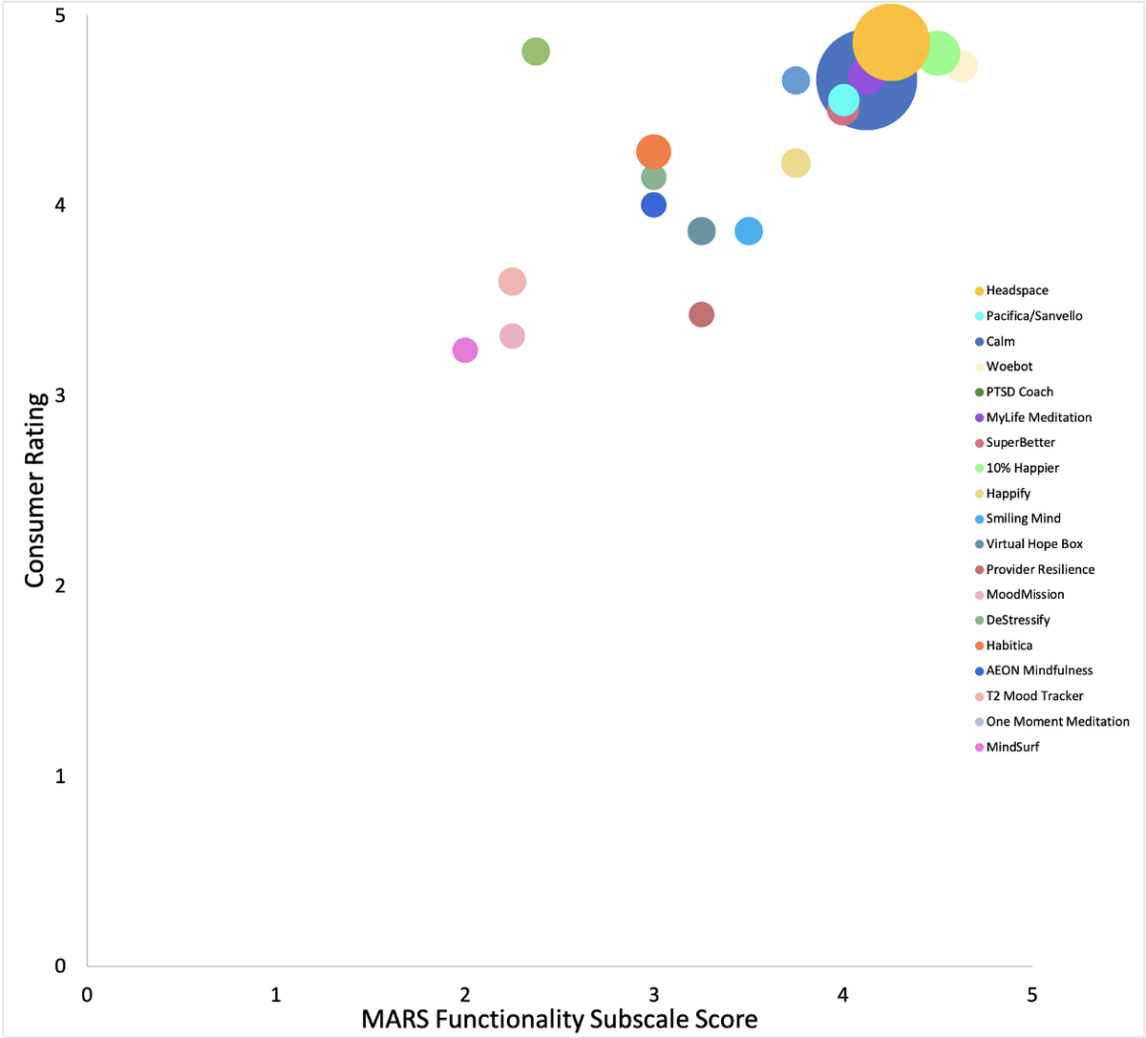
MARS Functionality subscale score × consumer rating scatterplot of 19 apps with variations in bubble size proportionate to app’s total number of consumer ratings. MARS: Mobile Application Rating Scale.

**Figure 2:**
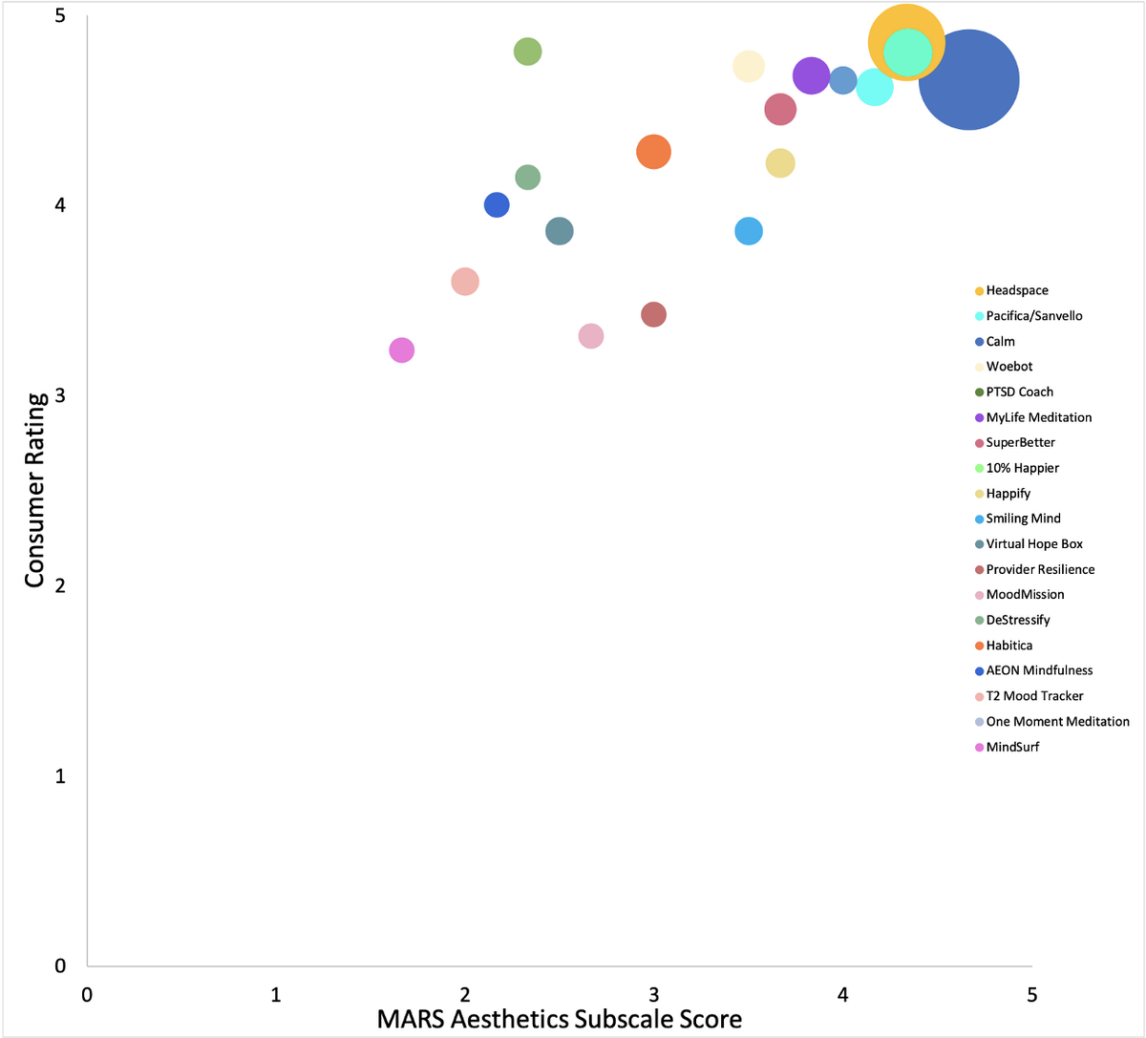
MARS Aesthetics subscale score × consumer rating scatterplot of 19 apps with variations in bubble size proportionate to app’s total number of consumer ratings. MARS: Mobile Application Rating Scale.

**Figure 3:**
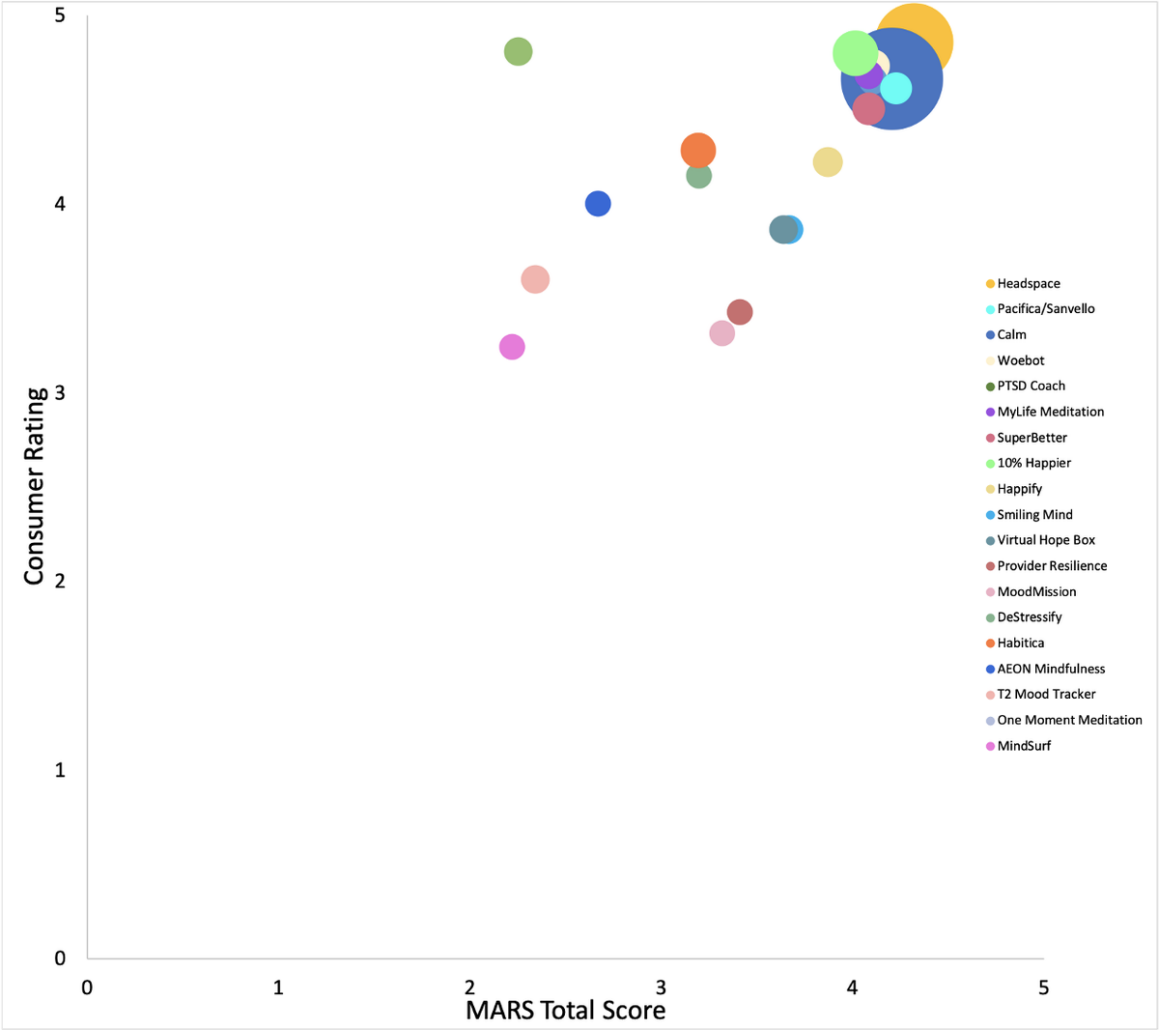
MARS total score × consumer rating scatterplot of 19 apps with variations in bubble size proportionate to app’s total number of consumer ratings. MARS: Mobile Application Rating Scale.

Each of the MARS subscales was significantly correlated with each other (r=0.63-0.88, *P*<.01) and the MARS total score (r=0.84-0.91, *P*<.001). Consumer ratings were correlated with the MARS Functionality subscale (r=0.74, *P*<.001; Figure 1), Aesthetics subscale (r=0.70, *P*<.01; Figure 2), and total score (r=0.58, *P*=.01; Figure 3). Number of evidence-based treatment components was not significantly associated with MARS scores (r=0.09, *P*=.73) or average consumer ratings (r=–.33, *P*=.17).

**Table 1:** MARS scores, consumer ratings, and number of evidence-based treatment components.

App name	MARS scores						Consumer ratings			Total number of evidence-based components
	Engagement	Functionality	Aesthetics	Information quality	Total score	Average user ratings		Total number of ratings		
10% Happier	3.80	4.50	4.33	3.43	4.02	4.80		96,707	3	
AEON Mindfulness	2.80	3.00	2.17	2.71	2.67	4.00		45	1	
Calm	4.60	4.13	4.67	3.43	4.21	4.66		1,431,242	1	
DeStressify	4.10	3.00	2.33	3.36	3.20	4.15		17	3	
Habitica	4.20	3.00	3.00	2.57	3.19	4.28		19,354	1	
Happify	4.50	3.75	3.67	3.57	3.87	4.22		5615	4	
Headspace	4.40	4.25	4.33	4.29	4.32	4.86		861,451	1	
MindSurf	3.00	2.00	1.67	2.21	2.22	3.24		18	4	
MoodMission	4.50	2.25	2.67	3.86	3.32	3.31		203	5	
One Moment Meditation	2.30	2.38	2.33	2.00	2.25	4.81		1281	1	
Pacifica/Sanvello	4.80	4.00	4.17	4.00	4.24	4.62		32,749	5	
Provider Resilience	3.40	3.25	3.00	4.00	3.41	3.43		43	2	
PTSD Coach	4.60	3.75	4.00	4.07	4.11	4.66		1781	3	
Smiling Mind	3.60	3.50	3.50	4.07	3.67	3.86		3600	2	
Stop, Breathe and Think/MyLife Meditation	4.60	4.13	3.83	3.79	4.09	4.68		39,329	1	
SuperBetter	4.60	4.00	3.67	4.07	4.00	4.50		12,737	1	
T2 Mood Tracker	2.40	2.25	2.00	2.71	2.34	3.60		1875	1	
Virtual Hope Box	4.80	3.25	2.50	4.00	3.64	3.86		1133	3	
Woebot	4.60	4.63	3.50	3.71	4.11	4.73		13,041	2	

## Discussion

### Principal Findings

In this paper, we described an analysis of the usability and quality of popular research-supported consumer mental health apps using the MARS which provides a bite-sized synthesis of app usability and quality that are easily accessible to consumers and researchers alike [1,18]. The mental health apps we evaluated were of good quality overall. We observed overall MARS scores that were comparable to other reviews of self-management apps [12,19,20]. Previous research has compared MARS scores with consumer ratings for various health-related apps; results varied in whether MARS scores were correlated with consumer ratings [8,9,12].

With regard to popular research-supported mental health apps, we draw the following conclusions: First, we found that consumer ratings were related to objective quality of the app overall. This suggests alignment between subjective assessment of quality by app users and objective assessment of quality by researchers. Second, consumer ratings were related to functionality and aesthetics. This suggests that design elements such as ease of use, navigation, graphics, and visual appeal may be more likely to impact the positivity of the user experience. Third, evidence-based treatment components were not associated with app quality or consumer ratings, and almost half of the apps had a singular skill focus. This suggests that quantity of evidence-based behavior change techniques designed and tested in traditional face-to-face mental health interventions is not what appeals to app consumers. Perhaps unimodal rather than multimodal intervention approaches lend themselves better to a self-guided format and decreases user burden.

### Limitations

This study did not evaluate all mental health apps available for public download, nor would it be feasible to do so. Rather, our analysis focused on a small targeted subset of apps identified in a prior publication that we utilized to address a new research question focused on objective and subjective app quality and user-centered design considerations for engagement and sustainability. Thus, our analysis may not represent the whole of all available resources. Apps were limited to English language that were available in the United States, downloadable on major app platforms, and with peer-reviewed publications.

### Conclusions and Implications

We found that objective app quality—and functionality and aesthetics in particular—was highly consistent with consumer appeal. Quantity of evidence-based—and presumably effective—behavior change techniques was not associated with app quality or consumer appeal. To translate evidence-based interventions to the competitive consumer app space, researchers should prioritize aspects of design that impact the user experience such as ease of use, navigation, graphics, and visual appeal. This work may be informed by user-centered design approaches in which iterative development of apps prioritize end user’s needs in the contexts in which the intervention will be implemented [21]. In addition, the complexity of evidence-based multimodal interventions may hinder chances of mHealth adoption. Adapting and optimizing design features to the individuals and settings that are unique to the digital space will help engage and retain users over time.

## Abbreviations

ICC: intraclass correlation coefficient
MARS: Mobile Application Rating Scale
